# Multilevel multivariate modeling on the association between undernutrition indices of under-five children in East Africa countries: evidence from recent demographic health survey (DHS) data

**DOI:** 10.1186/s40795-023-00741-w

**Published:** 2023-07-07

**Authors:** Abebew Aklog Asmare, Yitateku Adugna Agmas

**Affiliations:** 1 Department of Statistics, Mekdela Amba University, P.O. Box: 32, Tuluawlyia, Ethiopia; 2 Department of Rural Development and Agricultural Extension, Mekdela Amba University, P.O. Box: 32, Tuluawlyia, Ethiopia

**Keywords:** Prevalence, Multilevel multivariate binary logistic regression, East Africa, Stunting, Wasting, Underweight, Children aged under-five

## Abstract

**Background:**

Malnutrition is the main cause of illness and death in children under the age of five. It affects millions of children worldwide, putting their health and future in jeopardy. Therefore, this study aimed to identify and estimate the effects of important determinants of anthropometric indicators by taking into account their association and cluster effects.

**Method:**

The study was carried out in 10 countries in East Africa: Burundi, Ethiopia, Comoros, Uganda, Rwanda, Tanzania, Zimbabwe, Kenya, Zambia, and Malawi. A weighted total sample of 53,322 children under the age of five was included. Given the impact of other predictors such as maternal, child, and socioeconomic variables, a multilevel multivariate binary logistic regression model was employed to analyze the relationship between stunting, wasting, and underweight.

**Result:**

The study included 53,322 children, and 34.7%, 14.8%, and 5.1% were stunted, underweight, and wasted, respectively. Almost half of the children (49.8%) were female, and 22.0% lived in urban areas. The estimated odds of children from secondary and higher education mothers being stunted and wasted were 0.987; 95% CI: 0.979 – 0.994 and 0.999; 95% CI: 0.995 – 0.999, respectively, times the estimated odds of children from no education mothers. Children from middle-class families were less likely to be underweight than children from poorer families.

**Conclusion:**

The prevalence of stunting was higher than in the sub-Saharan Africa region, but the prevalence of wasting and underweight was lower. According to the study's findings, undernourishment among young children under the age of five continues to be a significant public health issue in the East African region. Governmental and non-governmental organizations should therefore plan public health participation focusing on paternal education and the poorest households in order to improve the undernutrition status of children under five. Additionally, improving the delivery of healthcare at health facilities, places of residence, children's health education, and drinking water sources are essential for lowering child undernutrition indicators.

## Introduction

Malnutrition is the main cause of illness and death in children under the age of five [1]. It affects millions of children worldwide, putting their health and future in jeopardy [2]. Even though the decline has not been consistent worldwide, its prevalence has decreased. In middle- and low-income countries, notably in Sub-Saharan Africa, child malnutrition is still an issue, and many children still suffer from chronic malnutrition [3, 4]. The most common symptom of chronic undernutrition in children is an inadequate intake of the nutrients and energy needed for growth and development [5]. Three anthropometric indices of malnutrition, stunting, underweight, and wasting, are used to evaluate nutritional deficiency or imbalance, the underlying causes of a number of children's health problems [6–8]. The three are well-known indicators of the severity of child malnutrition [9]. In poor countries, malnutrition contributes to more than half of under-five mortality. Sub-Saharan Africa has a very high proportion of under-five malnourished children, and daily death rates are rising [7].

Indicators of child malnutrition include stunting, wasting, and underweight, which refer to children who are, respectively, too short for their age (low height-for-age), too thin for their height (low weight-for-height), and too thin for their age (low weight-for-age). Height-for-age, weight-for-height, and weight-for-age z-scores are generated using the 2006 WHO child growth standards. Children who have a weight-for-height z-score (WHZ), a height-for-age z-score (HAZ), or a weight-for-age z-score (WAZ) below two are referred to as stunting, wasting, and underweight, respectively [6]. According to UNICEF, environmental, social, and economic factors all have a major impact on childhood malnutrition [8].

Each year, more than half of all children under the age of five die from malnutrition and its complications [10]. It has been established that undernourishment has a major negative impact on young children's growth and development [11]. The negative effects of childhood malnutrition are both short-term and long-term [12]. Long-term effects of undernutrition include poor educational practices [13], early mortality [11], and an increased risk of chronic illnesses like diabetes mellitus (DM), hypertension (HTN), and heart disease [14, 15]. Short-term effects of undernutrition include increased severity of illnesses [16], a delayed recovery period from disease [17], and delayed physical and mental development in children [18]. Additionally, undernutrition has a detrimental effect on female adolescents' reproductive health [19, 20]. Due to how frequently it occurs, undernutrition not only has detrimental impacts on a person's health but also negatively affects the economy. Through both direct production reduction owing to physical infirmity and indirect cognitive dysfunction and educational impairments, this condition limits economic growth and perpetuates poverty. In addition, a poor diet raises the cost of medical care [21].

In 2020, undernutrition was a factor in about half of the cases of childhood mortality [22]. In the same year, 12.6% of children under the age of five are underweight, 6.7% are wasted, and 22% are stunted [22]. Around 149.2 million children under the age of five were affected by stunting in 2020, with 53% of these children residing in Asia and 30.7% in Africa [22]. 45.4 million Children under the age of five perished because of waste. More than two-thirds of all wasted children are located in Asia, and more than a quarter are found in Africa [22]. Stunting occurs more frequently in Eastern Africa (32.6%) than in Western Africa (30.9%), Northern Africa (21.4%), or Southern Africa (23.3%), according to a more thorough analysis of the distribution of undernutrition on the African continent [22].

Numerous factors contribute to childhood stunting, wasting, and being underweight. The collective causes reported by several studies include the age of the child in a month [23–26], the gender of the child [27, 28], the birth size of children [29, 30], the birth order of the child [31, 32], age of mother at first birth and the maternal education [32, 33], household wealth index [23, 31, 34], the source of drinking water [24, 34], the family size [27], the place of residence [27], the husband’s education level [31, 34], breastfeeding [35, 36], diarrhea [36, 37], fever [35] and cough [36, 38] in the last two weeks prior to the survey, the birth type of child [24, 32], number of children aged under-five [30] have been identified as some of the factors of childhood undernutrition status in East Africa.

Numerous studies on stunting, underweight, and wasting were carried out in East Africa. However, they paid little attention to their relationship and were unable to identify the associated determinants of anthropometric indicators among East African children under the age of five. In a study carried out in Ethiopia, India, and Malawi [9, 39, 40], the link between undernutrition indices was examined. The association was determined without taking into account the impact of additional factors linked to stunting, underweight, and wasting. Other East African and international researchers examined each indicator of undernutrition separately and determined the corresponding factors [32, 41–44]. However, it was noted that there is a dearth of research available or undertaken in East Africa that identifies and estimates the drivers of undernutrition indicators in children under the age of five, such as stunting, underweight, and wasting, by taking into account the relationships between them. Furthermore, despite the fact that the population of East Africa is not uniform in terms of its culture, language, and other characteristics, the cluster effect receives little consideration. When there is a chance that the outcomes of patients in comparable groups may correlate, clustering effects may develop, which may lead to a loss of observational independence [45]. Children from the same nation are more likely to have similar undernutrition status than children from different countries; therefore, when the cluster effect is ignored, the key determinants of a child's undernutrition status within and between countries (cluster) are inadequately taken into account. As a result, the aim of this study was to identify and estimate the influence of significant factors on indicators of undernutrition.

## Method

Data from the Demographic and Health Survey (DHS), which was conducted using a cross-sectional study methodology, were used in this investigation. For this particular study, we used Kids Record (KR) files, which include data on moms and children. We predict that children in the same cluster will be more similar than children across the country because the DHS data had a hierarchical structure and children were nested within clusters. The heterogeneity between clusters should therefore be taken into consideration using sophisticated models. A mixed-effect logistic regression model was therefore built (with both fixed and random effects).

### Data source and sampling method

The current study is based on data from the most recent Demographic and Health Surveys (DHS) conducted in ten East African countries (Burundi, Ethiopia, Comoros, Uganda, Rwanda, Tanzania, Zimbabwe, Kenya, Zambia, and Malawi). All datasets were combined to determine the region's pooled prevalence and related causes of undernutrition indicators among children aged under-five. The DHS survey employed stratified, two-stage cluster sampling. The data was obtained from the Measure DHS website, which can be found at https://dhsprogram.com/Data/terms-of-use.cfm. Variables in this study (both dependents and independents) were taken from the Kid Record (KR file) data set after approval was granted via an online request stating the objective of this study. The current study used a weighted total sample of 53,322 children under the age of five.

### Inclusion/exclusion criteria

Children under the age of five who completed relevant forms about personal information and clinical signs met the inclusion criteria. As a result, children who had not completed all relevant information (questionaries’) or who were over the age of five were excluded.

### Study variables

#### Response variables

Once the ${Z}_{i}$ for each child is calculated, the undernutrition indices were recoded into dichotomies variables as:

- stunted $$(0 = No\,if\,HAZ \ge - 2\,and\,1 = Yes\,if\,HAZ < - 2)$$,
- wasted $$(0 = No\,if\,WHZ \ge - 2\,and\,1 = Yes\,if\,WHZ < - 2)$$, and
- underweight $$(0 = No\,if\,WAZ \ge - 2\,and\,1 = Yes\,if\,WAZ < - 2)$$  

The outcome variables were measured using WHO 2006 child growth standards [46].

#### Independent variables

The independent variables were chosen based on previous research on factors influencing children's undernutrition status [47–49]. These variables are created by combining naturally continuous and discrete variables into categories. Table 3 lists the independent variables associated with the three-undernutrition indicators and reflected in this study. Face-to-face interviews with mothers and caregivers were used to collect the characteristics listed in Table 3.

### Data management and analysis

The variables were extracted from the literature, and then we integrated the DHS data from the 10 East African nations. Before performing any statistical analysis, the data were weighted to restore survey representativeness and take into consideration the sample design for generating standard errors and trustworthy estimates. This was done using sampling weight, a primary sampling unit, and strata. R 4.2.2 was employed for the cross-tabulations and summary statistics. In order to find relevant factors connected to undernutrition indicators, a multilevel, multivariate logistic model was utilized. Adjusted odds ratios (AOR) with a 95% confidence interval (CI) and a p-value less than or equal to 0.05 were used.

In present study, let ${Y}_{1i}, {Y}_{2i} and {Y}_{3i}$ are the dichotomies outcome of stunting, underweight, and wasting of the ${i}^{th}$ under-five years children, respectively. For dichotomies outcome ${Y}_{ji}$ and a vector of independent variables X, multivariate binary logistic regression model is given by [50]:

1 $${\pi }_{j\left(X\right)}=\frac{{e}^{{\beta }_{j0}+{\beta }_{j1}{X}_{1}+{\beta }_{j2}{X}_{2}+\dots +{\beta }_{jp}{X}_{p}}}{{1+e}^{{\beta }_{j0}+{\beta }_{j1}{X}_{1}+{\beta }_{j2}{X}_{2}+\dots +{\beta }_{jp}{X}_{p}}}=\frac{{e}^{X{\beta }_{j}}}{1+{e}^{X{\beta }_{j}}},j=\mathrm{1,2},3$$

Where ${\pi }_{j(X)}=P({Y}_{ji}={}^{1}\!\left/ \!{}_{X}\right.)$, the probability of the ${i}^{th}$ children aged under-five being stunted (${Y}_{1i}$), underweight (${Y}_{2i}$), and wasting (${Y}_{3i}$) given other predictors X. Regularly, the logit (log odds) that marked linear association with independent variables can be stated as:

2 $$logit\left[P\left({Y}_{ji}={}^{1}\!\left/ \!{}_{X}\right.\right)\right]={\beta }_{j0}+{\beta }_{j1}{X}_{1}+{\beta }_{j2}{X}_{2}+\dots +{\beta }_{jp}{X}_{p}={X\beta }_{j}, j=\mathrm{1,2},3$$

The odds ratio is the best method that is used to measure the relationship between categorical variables in the logistic regression model. It is the proportion of odds defined as:

3 $${OR}_{j}=\frac{{}^{{\pi }_{j}({X}_{1})}\!\left/ \!{}_{1-{\pi }_{j}({X}_{1})}\right.}{{}^{{\pi }_{j}({X}_{2})}\!\left/ \!{}_{1-{\pi }_{j}({X}_{2})}\right.}$$

### Multilevel multivariate logistic regression

Separate evaluations of anthropomorphic indicators, including underweight, stunting, and wasting, in children under the age of five were carried out in several studies [6, 25, 27, 29, 30, 51]. In this instance, logistic regression analysis is sufficient for determining the covariate's impact on the dependent variable. The relationship between the anthropomorphic indicators, however, would not be taken into account in a separate analysis. It is more logical to account for the relationship between estimates of covariate effects and anthropometric indices using a multivariate logistic regression model [49]. It is used to simultaneously model several interesting categorical outcomes and analyze how they relate to other factors [47, 52].

To quantify the impact of variables on anthropometric measures in a nation with a varied population, like East Africa, a multivariate logistic regression model is insufficient. According to DHS data gathered from children living in different nations in East Africa, there is a very high probability of a clustering effect. If there is a clustering effect in the dataset, the results will be disorganized. The intraclass correlation coefficient (ICC) [47, 53] and median odds ratio (MOR) [54] were used to assess the clustering effect. The ICC can be calculated using the formula shown below:

4 $$ICC=\frac{{\widehat{\sigma }}_{r}^{2}}{{\widehat{\sigma }}^{2}+{\widehat{\sigma }}_{r}^{2}}$$

where ${\widehat{\sigma }}_{r}^{2}$ and ${\widehat{\sigma }}^{2}$ are the estimated cluster variance (regarding to country) and residual variance, respectively [47, 53]. The $MOR$ is defined as: $MOR=exp\left[0.6745\sqrt{2{\widehat{\sigma }}_{r}^{2}}\right]$ [47, 52]. After arranging the data in SPSS 26, the statistical analysis was carried out using R software, VGAM, and the GLMER package.

Prior to fitting the model, it is necessary to assess the model's suitability or goodness of fit. The evaluated model's forecasting ability may help to understand this. In a multivariate logistic regression model, the concordance proportion is frequently used to assess or identify the predictive power. The concordance value calculates the likelihood that the predictions and results are in agreement, or whether the anticipated outcome matches the actual outcome [50]. In order to assess how well the projected model approximates the data, the concordance proportion was evaluated in this study.

## Results

### Characteristics of the outcome variables

This study included a weighted total of 53,322 children aged under-five, with 34.7% (18,527), 14.8% (7894), and 5.1% (2743) suffering from stunting, underweight, and wasting, respectively (Table 1). The prevalence of stunting, underweight, and wasting was different across countries. The prevalence of stunting, wasting, and underweight was lowest in Zimbabwe (25.3%), Rwanda (2.3%), and Zimbabwe (7.5%), respectively, whereas the prevalence of stunting, wasting, and underweight was highest in Burundi (55.3%), the Comoros (11.0%), and Burundi (286.6%), respectively (Table 2).

**Table 1:** Anthropometric outcomes of the sample (*n* = 53,322)

Outcome variables	Categories	Percentage (95%CI)
Stunting	Yes	34.7 (34.30 – 35.10)
Underweight	Yes	14.8 (14.50 – 15.10)
Wasting	Yes	5.1 (4.91 – 5.29)

**Table 2:** Frequency distribution of stunting, underweight and wasting in East Africa

Region	Country Name	DHS year	Total frequency (%)	Stunting	Underweight	Wasting
				**Yes (%)**	**Yes (%)**	**Yes**
**East Africa**	Burundi	2016/17	5596 (10.5)	3097 (55.3)	1601 (28.6)	281 (5.0)
	Comoros	2012	2087 (3.9)	611 (29.3)	321 (15.4)	230 (11.0)
	Ethiopia	2016	8876 (16.6)	3384 (38.1)	2052 (23.1)	887 (10.0)
	Kenya	2014	7554 (14.2)	1950 (25.8)	806 (10.7)	281 (3.7)
	Malawi	2015/16	4262 (8.0)	1553 (36.4)	464 (10.9)	112 (2.6)
	Rwanda	2019/20	3266 (13.7)	1226 (37.5)	287 (8.8)	74 (2.3)
	Tanzania	2015/16	7294 (13.7)	2453 (33.6)	974 (13.4)	341 (4.7)
	Uganda	2016	3563 (1.9)	1002 (28.1)	341 (9.6)	120 (3.4)
	Zambia	2018	6342 (11.9)	2118 (33.4)	711 (11.2)	263 (4.1)
	Zimbabwe	2015	4481 (8.4)	1133 (25.3)	337 (7.5)	154 (3.4)

### Characteristics of the Independent variables

The result of Table 3 revealed that more than half (50.2%) of children aged under-five were males, and 79.2% of them were in the age group of 12 to 59 months. More than half of the mothers (57.5%) had their first child when they were under the age of 20. 45.4% of children aged under-five were born into poor households. More than three-fourths of (78.0%) householders were rural settlers. The overall prevalence of stunting, underweight, and wasting among children aged under-five in the region was 34.7, 14.6, and 5.1%, respectively.

**Table 3:** Independent variable description and frequency distribution in East Africa

Variables	Categories	Weighted frequency (%)
Place of residence	Urban	11,745 (22.0)
	Rural	41,577 (78.0)
Education level of mother	No education	14,224 (26.7)
	Primary	26,393 (49.5)
	Secondary and above	12,075 (23.8)
Source of drinking water	Not improved	36,528 (68.5)
	Improved	16,794 (31.5)
Family members	Small	15,810 (29.7)
	Medium	33,167 (62.2)
	Large	4345 (8.1)
Number of children aged under-five	Only one	19,726 (37.0)
	Two	24,135 (45.3)
	Three and more	9461 (17.7)
Wealth index	Poor	24,183 (45.4)
	Middle	10,462 (19.6)
	Rich	18,677 (35.0)
Mother age at first birth	Less than 20	30,643 (57.5)
	20 to 34	22,559 (42.3)
	35 to 49	120 (0.2)
Husband education level	No education	10,585 (19.9)
	Primary	25,959 (48.7)
	Secondary and above	16,778 (31.5)
Birth order of child	First	10,343 (19.4)
	2 to 3	19,880 (37.3)
	4 to 5	12,361 (23.2)
	6 and more	10,737 (20.1)
Birth type of child	Single birth	51,908 (97.3)
	Multiple births	1414 (2.7)
Sex of child	Male	26,770 (50.2)
	Female	26,552 (49.8)
Breastfeeding	No	953 (1.8)
	Yes	52,369 (98.2)
Place of delivery	Home	17,099 (32.1)
	Health facility	36,223 (67.9)
Size of children at birth	Small	8437 (15.8)
	Average	29,246 (54.8)
	Large	15,639 (29.3)
Diarrhea	No	44,737 (83.9)
	Yes	8585 (16.1)
Fever	No	41,230 (77.3)
	Yes	12,091 (22.7)
Cough	No	38,265 (71.8)
	Yes	15,057 (28.2)
	Infant	11,107 (20.8)
	12 to 59	42,215 (79.2)

The results in Tables 4 and 5 indicated that children under five were affected by more than one of the three undernutrition indicators; as a result, consideration is essential in empathetically treating the total number of malnourished children. For example, 5689 (10.67%) of the children were both stunted and underweight. For more detail, see the results in Tables 4 and 5.

**Table 4:** Simultaneous frequency distribution of stunting, underweight and wasting

				**Underweight**		**Total**
				No	Yes	
	No	**Stunting**	No	32,448	456	32,904
**Wasting**			Yes	11,986	5,689	17,675
	Yes	**Stunting**	No	994	897	1,891
			Yes	0	853	853
**Total**				45,428	7,895	53,323

**Table 5:** Cross-classification of undernutrition indicators and resultant frequency distribution

Undernourished indicators	Frequency (%)
Non- undernourished	32,448 (60.85)
Stunting only	11,986 (22.48)
Underweight only	456 (0.86)
Wasting only	994 (1.86)
Stunting and underweight	5, 689 (10.67)
Stunting and wasting	0 (0.0)
Underweight and wasting	897 (1.68)
Stunting, underweight and wasting	853 (1.60)

The whole prevalence of indicators of undernourishment was measured by the composite index of anthropometric failure (CIAF) [55]. Accordingly, children aged under-five can be grouped into eight categories: no failure; stunted only; underweight only; wasted only; stunted and underweight; underweight and wasted; and stunted, underweight, and wasted. According to the composite index of failure, approximately 39.15% = (22.48 + 0.86 + 10.67 + 1.68 + 0.60) % of children aged under-five were diagnosed with malnutrition, whereas 60.85% of the children sampled in East Africa were not diagnosed with malnutrition. The result indicates that 39.15% of children were stunted, underweight, or wasted. This suggests that the occurrence of overall undernourishment in children is about 39.15% in the region.

Table 6 shows all of the possible pairwise relationships between stunting, underweight, and wasting using odds ratios (OR). The odds ratios for the dependency among stunting and underweight, stunting and wasting, and underweight and wasting were 3.566, 1,121, and 2.941, respectively. The result is different from unity. This indicates a dependency between the three anthropometric indicators, and hence fitting a multivariate logistic model for the three dependents is suitable to include their dependency and estimate the effects of the predictors.

**Table 6:** Pairwise dependency between undernutrition indicators using odds ratio (OR)

	**Stunting**	**Underweight**
	**OR ((95% CI)**	**OR ((95% CI)**
**Wasting**	1.121 (0.879 – 1.412)	2.941 (2.872 – 3.011)
**Underweight**	3.566 (3.054 – 3.328)	

Table 7 shows the bivariate analysis of the relationship between independent variables and each of the undernourishment indicators and the distribution of scores below five at each level of the independent variables. Place of residence, education level of mother, source of drinking water, family size, number of children aged under-five, wealth index, age of mother at first birth, education level of husband, birth order of children, birth type of children, sex of children, place of delivery, and size of children at birth were independent variables that were independently associated (*p*-value 0.05) with the three undernourishment indicators.

**Table 7:** Bivariate Analysis of stunting, underweight, and wasting with the predictors

Predictors	Stunting		*P*-value	Underweight		*P*-value	Wasting		*P*-value
	No (%)	Yes (%)		No (%)	Yes (%)		No (%)	Yes (%)	
Place of residence									
Urban	8893 (75.7)	2852 (24.3)	0.000	10,747 (91.5)	997 (8.5)	0.000	11,225 (95.6)	520 (4.4)	0.000
Rural	25,902 (62.3)	15,676 (37.3)		34,681 (83.4)	6897 (16.6)		39,354 (94.7)	2223 (5.3)	
Education level of mother									
No education	8072 (56.7)	6152 (43.3)		10,852 (76.3)	3372 (23.7)		13,087 (92.0)	1137 (8.0)	
Primary	16,908 (64.1)	9484 (35.9)	0.000	22,833 (86.5)	3560 (13.5)	0.000	25,294 (95.8)	1099 (4.2)	0.000
Secondary +	9815 (77.2)	2891 (22.8)		11,744 (92.4)	962 (7.6)		12,198 (96.0)	507 (4.0)	
Source of drinking water									
Not improved	23,473 (64.3)	13,058 (35.7)	0.000	31,044 (85.0)	5488 (15.0)	0.045	34,588 (94.7)	1940 (5.3)	0.010
Improved	11,322 (67.4)	5472 (32.6)		14,384 (85.6)	2410 (14.4)		15,990 (95.2)	803 (4.8)	
Family size									
Small	10,431 (66.0)	5380 (34.0)		13,740 (86.9)	2071 (13.1)		15,088 (95.4)	723 (4.6)	
Medium	21,433 (64.6)	11,734 (35.4)	0.000	27,976 (84.3)	5191 (15.7)	0.000	31,408 (94.7)	1759 (5.3)	0.000
Large	2931 (67.5)	1414 (32.5)		3712 (85.4)	633 (14.6)		4083 (94.0)	261 (6.0)	
Number of under-five children									
Only one	13,433 (68.1)	6292 (31.9)		17,250 (87.4)	2476 (12.6)		18,841 (95.5)	885 (4.5)	
Two	15,288 (63.1)	8907 (36.9)	0.000	20,276 (84.0)	3859 (16.0)	0.000	22,892 (94.8)	1243 (5.2)	0.000
3 and more	6133 (64.8)	3328 (35.2)		7902 (83.5)	1559 (16.5)		8846 (93.5)	615 (6.5)	
Wealth index									
Poor	14,355 (59.4)	9828 (40.6)		19,645 (81.2)	4539 (18.8)		22,747 (94.1)	1437 (5.9)	
Middle	6676 (63.8)	3786 (36.2)	0.000	8893 (85.0)	1568 (15.0)	0.000	9915 (94.8)	547 (5.2)	0.000
Rich	13,763 (73.7)	4913 (26.3)		16,890 (90.4)	1787 (9.6)		17,917 (95.9)	759 (4.1)	
Mother age at first birth									
Less than 20	19,658 (64.2)	10,985 (35.8)		26,114 (85.2)	4529 (14.8)		29,132 (95.1)	1511 (4.9)	
20 to 34	15,064 (66.8)	7495 (33.2)	0.000	19,221 (85.2)	3337 (14.8)	0.031	21,336 (94.6)	1223 (5.4)	0.021
35 to 49	72 (60.0)	48 (40.0)		92 (76.7)	28 (23.3)		111 (92.5)	9 (7.5)	
Education level of husband									
No education	5903 (55.8)	4681 (44.2)		7982 (75.4)	2602 (24.6)		9707 (91.7)	878 (8.3)	
Primary	16,442 (63.3)	9517 (36.7)	0.000	22,159 (85.4)	3800 (14.6)	0.000	24,798 (95.5)	1161 (4.5)	0.000
Secondary +	12,449 (74.2)	4329 (25.8)		15,287 (91.1)	1491 (8.9)		16,074 (95.8)	704 (4.2)	
Birth order of child									
First	6929 (67.0)	3414 (33.0)		9040 (87.4)	1303 (12.6)		9871 (95.4)	473 (4.6)	
2 to3	13,407 (67.4)	6473 (32.6)	0.000	17,317 (87.1)	2563 (12.9)	0.000	18,964 (95.4)	917 (4.6)	0.000
4 to 5	7881 (63.8)	4480 (36.2)		10,318 (83.5)	2043 (16.5)		11,663 (94.4)	698 (5.6)	
6 and more	6577 (61.2)	4161 (38.8)		8752 (81.5)	1985 (18.5)		10,082 (93.9)	656 (6.1)	
Birth type of child									
Single birth	34,131 (65.8)	17,777 (34.2)	0.000	44,405 (85.5)	7503 (14.5)	0.000	49,260 (94.9)	2648 (5.1)	0.007
Multiple births	663 (46.9)	751 (53.1)		1023 (72.3)	391 (27.7)		1319 (93.3)	95 (6.7)	
Sex of child									
Male	16,691 (62.3)	10,079 (37.3)	0.000	22,567 (84.3)	4203 (15.7)	0.000	25,304 (94.5)	1466 (5.5)	0.000
Female	18,103 (68.2)	8449 (31.8)		22,861 (86.1)	3691 (13.9)		25,275 (95.2)	1277 (4.8)	
Breastfeeding									
No	582 (61.1)	370 (38.9)	0.007	757 (79.4)	196 (20.6)	0.000	891 (93.6)	61 (6.4)	0.075
Yes	34,212 (65.3)	18,157 (34.7)		44,671 (85.3)	7698 (14.7)		49,687 (94.9)	2682 (5.1)	
Place of delivery									
Home	10,434 (61.0)	6665 (39.0)	0.000	13,641 (79.8)	3458 (20.2)	0.000	15,889 (92.9)	1210 (7.1)	0.000
Health facility	24,361 (67.3)	11,863 (32.7)		31,787 (87.8)	4436 (12.2)		34,690 (95.8)	1533 (4.2)	
Size of children at birth									
Small	4688 (55.6)	3749 (44.4)		6315 (74.8)	2122 (25.2)		7734 (91.7)	704 (8.3)	
Average	19,361 (66.2)	9885 (33.8)	0.000	25,200 (86.2)	4046 (13.8)	0.000	27,847 (95.2)	1399 (4.8)	0.000
Large	10,745 (68.7)	4893 (31.3)		13,913 (89.0)	1726 (11.0)		14,998 (95.9)	641 (4.)	
Diarrhea									
No	29,476 (65.9)	15,261 (34.1)	0.000	38,410 (85.9)	6327 (14.1)	0.000	42,524 (951.)	2212 (4.9)	0.000
Yes	5319 (62.0)	3266 (38.0)		7018 (81.7)	1567 (18.3)				
Fever									
No	27,285 (66.2)	13,946 (33.8)	0.000	35,464 (86.0)	5767 (14.0)	0.000	39,195 (95.1)	2035 (4.9)	0.000
Yes	7510(62.1)	4582 (37.9)		9964 (82.4)	2127 (17.6)		11,384 (94.1)	708 (5.9)	
Cough									
No	24,970 (65.3)	13,295 (34.7)	0.982	32,615 (85.2)	5650 (14.8)	0.686	36,250 (94.7)	2015 (5.3)	0.043
Yes	9824 (65.2)	5233 (34.8)		12,813 (85.1)	2244 (14.9)		14,328 (95.2)	728 (4.8)	
Age of children in months									
Infant	9138 (82.3)	1969 (17.7)	0.000	9977 (89.8)	1130 (10.2)	0.000	10,228 (92.1)	879 (7.9)	0.000
12 to 59	25,657 (60.8)	16,559 (39.2)		35,451 (84.0)	6764 (16.0)		40,351 (95.6)	1864 (4.4)	

### Parameter estimation

#### Random effect model

The calculated ICCs for stunting, underweight, and wasting were 59.1%, 55.87%, and 56.61%, respectively, which is a high value. This strongly suggests that there is a clustering effect and that there is between-group variability that would benefit from a cluster effect due to country. A high ICC, in this sense, indicates a high degree of similarity among children from the same country who have undernutrition indicators. On the other hand, the estimated clustering variance on stunting (${\widehat{\sigma }}_{rs}^{2}$), underweight (${\widehat{\sigma }}_{ru}^{2}$) and wasting (${\widehat{\sigma }}_{rw}^{2}$) found to be significant (p-value 0.05) in the model indicates that there is a country effect in the model. Stunting (MOR = 1.593), underweight (MOR = 1.492), and wasting (MOR = 1.515) have different MOR values. This indicates that there is significant clustering variation. To take into account the cluster effect because of country, a multilevel multivariate logistic regression model was applied because the traditional model cannot remove the cluster effect because the traditional model cannot remove the cluster effect (see Table 8).

**Table 8:** Parameter estimation of undernutrition indicators using Multivariate binary logistic regression

Predictors	Stunting		Underweight		Wasting	
	AOR (95% CI)	*P*-value	AOR (95%CI)	*P*-value	AOR (95%CI)	*P*-value
Intercept	3.188 (3.054, 3.328)	0.000	2.941 (2.872, 3.011)	0.000	2.765 (2.741, 2.790)	0.000
Place of residence						
Urban	Ref		Ref		Ref	
Rural	1.052 (1.040, 1.064)	0.000	1.006 (1.000, 1.013)	0.000	1.000 (0.997, 1.001)	0.416
Education level of mother						
No education	Ref		Ref		Ref	
Primary	0.992 (0.912, 0.931)	0.000	0.974 (0.968, 0.979)	0.000	0.998 (0.995, 1.000)	0.033
Secondary +	0.987 (0.979, 0.994)	0.000	1.000 (0.999, 1.013)	0.078	0.999 (0.995, 0.999)	0.018
Source of drinking water						
Not improved	1.022 (1.012, 1.031)	0.000	1.007 (1.002, 1.012)	0.007	1.000 (0.998,1.001)	0.656
Improved	Ref		Ref		Ref	
Family size						
Small	0.969 (0.957, 0.982)	0.000	0.995 (0.988, 1.003)	0.207	1.000 (0.997, 1.002)	0.740
Medium	0.992 (0.984, 1.000)	0.061	0.997 (0.992, 1.001)	0.129	1.000 (0.998, 1.017)	0.768
large	Ref		Ref		Ref	
Number of under-five children						
Only one	1.007 (0.998, 1.017)	0.135	1.001 (0.995, 1.006)	0.836	1.001 (0.999, 1.003)	0.379
Two	0.969 (0.962, 0.976)	0.000	0.993 (0.989, 0.997)	0.000	1.000 (0.999, 1.002)	0.926
3 and more	Ref		Ref		Ref	
Wealth index						
Poor	Ref		Ref		Ref	
Middle	0.944 (0.936, 0.951)	0.000	0.986 (0.982, 0.990)	0.000	0.999 (0.997, 1.001)	0.294
Rich	0.987 (0.978, 0.996)	0.004	1.000 (0.995, 1.005)	0.979	1.000 (0.998, 1.002)	0.797
Mothers at first birth						
Less than 20	Ref		Ref		Ref	
20 to 34	1.049 (0.986, 1.116)	0.129	1.018 (0.984, 1.053)	0.307	1.001 (0.988, 1.014)	0.876
35 to 49	1.019 (0.983, 1.057)	0.295	1.003 (0.983, 1.023)	0.771	1.000 (0.993, 1.008)	0.986
Husband education level						
No education	Ref		Ref		Ref	
Primary	0.951 (0.941, 0.961)	0.000	0.976 (0.970, 0.981)	0.000	0.998 (0.996, 1.000)	0.063
Secondary +	0.994 (0.987, 1.002)	0.124	1.001 (0.998, 1.015)	0.000	1.002 (0.999, 1.003)	0.009
Birth order of children						
First	Ref		Ref		Ref	
2 to 3	1.000 (0.989, 1.011)	0.996	1.002 (0.996, 1.008)	0.486	1.000 (0.998, 1.002)	0.951
4 to 5	1.004 (0.996, 1.013)	0.326	1.001 (0.996, 1.006)	0.770	1.000 (0.999, 1.002)	0.649
6 and more	0.990 (0.982, 0.998)	0.011	0.996 (0.992, 1.001)	0.059	1.000 (0.998, 1.001)	0.078
Birth type of children						
Single birth	Ref		Ref		Ref	
Multiple births	1.272 (1.240, 1.305)	0.000	1.069 (1.054, 1.084)	0.000	1.001 (0.996, 1.007)	0.610
Sex of children						
Male	Ref		Ref		Ref	
Female	0.937 (0.929, 0.944)	0.000	0.990 (0.985, 0.994)	0.000	0.998 (0.997, 1.000)	0.064
Breastfeeding						
No	Ref		Ref		Ref	
Yes	0.999 (0.992, 1.071)	0.078	0.993 (0.977, 1.009)	0.391	0.997 (0.991, 1.003)	0.290
Place of delivery						
Home	Ref		Ref		Ref	
Health facility	0.998 (1.022, 1.042)	0.000	0.985 (0.980, 0.990)	0.000	0.997 (0.995, 0.999)	0.002
Birth size of a child						
Small	Ref		Ref		Ref	
Average	0.904 (0.896, 0.912)	0.000	0.956 (0.951, 0.961)	0.000	0.996 (0.994, 0.998)	0.000
Large	1.027 (1.020, 1.034)	0.000	1.015 (1.011, 1.019)	0.000	1.002 (1.000, 1.003)	0.029
Diarrhea						
No	Ref		Ref		Ref	
Yes	1.048 (1.036, 1.060)	0.000	1.017 (1.010, 1.023)	0.000	1.001 (0.999, 1.004)	0.239
Fever						
No	Ref		Ref		Ref	
Yes	1.022 (1.011, 1.033)	0.000	1.010 (1.004, 1.016)	0.000	1.001 (0.999, 1.003)	0.346
Cough						
No	Ref		Ref		Ref	
Yes	1.003 (0.993, 1.013)	0.563	1.000 (0.995, 1.006)	0.992	0.999 (0.997, 1.001)	0.298
Age of children in months						
Infant	Ref		Ref		Ref	
12 to 59	1.166 (1.157, 1.174)	0.000	1.020 (1.016, 1.024)	0.000	1.002 (1.002, 1.017)	0.000
**Random effect**						
Variance	2.790	0.000	2.446	0.000	2.521	0.000
ICC%	59.10		55.87		56.61	
**Dependency**		**OR (95%CI)**		*P***-value**		
Stunting and underweight		3.188 (3.054 – 3.328)		0.000		
Stunting and wasting		1.015 (0.733 – 1.406)		0.136		
Underweight and wasting		2.734 (2.734 – 2.879)		0.000		

Key: *Ref.* Reference, *OR* Odds ratio, *CI* Confidence Interval

#### Fixed effect model

The independent variables that were included in the current study were: the education level of the mother, place of delivery, birth size of children, age of children, and the husband's education level. These common predictors were significantly associated with the three undernourishment indices. Fever and diarrhea in the two weeks prior to the survey, gender of children, birth type of children, wealth status of the household, number of children aged under-five in the household, source of drinking water, and place of residence were common determinants that were significantly associated with both stunting and underweight. Where family size and birth order of children were predictors that were significantly associated with stunting (Table 8).

A multilevel multivariate binary logistic regression model for estimation of undernourishment indicators was used to calculate concordant and discordant indices. 79.8% of children under the age of five with undernourishment indicators such as stunting, underweight, and waste would have a good chance of estimating their stunting, underweight, and waste levels. In this respect, the concordant index was very high. Indicating that the model's ability to explain the association between the indicators was very decent and fit the data well.

## Discussion

In this study, the impacts of the independent variables were assessed using data from a recent demographic health survey in east Africa to examine the association between undernutrition indicators such as stunting, underweight, and wasting in children under the age of five. This research discovered a strong link between stunting, wasting, and underweight status. Furthermore, it was discovered that underweight is a composite measure of stunting and wasting when the relationship between the three indicators is looked at, even when the impacts of other independent variables are not taken into consideration. Previous studies from Ethiopia, India, and Malawi [9, 39, 40] support this conclusion.

In this study, the prevalence of stunting, underweight, and wasting were 34.7, 14.8, and 5.1%, respectively. The prevalence of wasting and underweight were lower than the study reported in sub-Saharan Africa [3], but higher than the study reported in India [39], whereas the prevalence of stunting was higher than the study reported in sub-Saharan Africa [3]. This is due to the fact that the region is clearly affected by food scarcities, harsh climatic conditions, and drought situations, as well as inadequate access to land for farming purposes [56]. These dynamics extremely challenge progress toward improving farming efficiency, nutrition security, and child nutrition in east Africa.

The mother's education level, the location of the delivery, the size at birth and age of the children, as well as the husband's education level, were found to be the common predictors that were significantly linked to the three undernourishment indices. This is in line with earlier research in Sub-Saharan Africa and Ethiopia [24, 43]. The likelihood of children being stunted, underweight, or wasted decreased as father education increased, which was consistent with research conducted in Ethiopia [57]. Children born in medical institutions had a lower chance of being stunted, underweight, or wasted. Which agreed with the results of a study conducted in Sub-Saharan Africa [46]. A child with a small birth size has a higher risk of developing malnutrition than a child with an average birth size. Which was in line with the research in Ethiopia and Sub-Saharan Africa [46, 57]. According to a recent study, a child's nutritional indicators were highly correlated with the child's age. This result is in line with research conducted in Burkina Faso, Ethiopia, and Bangladesh [26, 58–60], which discovered that a child's risk of malnutrition rose with age. The late introduction of supplemental foods with poor nutritional value is one potential cause [61].

Common determinants that were significantly associated with stunting and underweight included fever and diarrhea in the two weeks prior to the survey, the gender of the children, their birth type, the wealth status of the household, the number of children under the age of five living in the household, the source of drinking water, and their place of residence, which is consistent with the findings in Ethiopia [24]. According to results from research in Gahanna and Pakistan [36, 37], children who had a fever two weeks prior to the survey period had a higher risk of stunting and underweight than children who had no fever at the time. Children who experienced diarrhea two weeks before the survey were more likely to be stunted and underweight than those who did not. This outcome is congruent with what was discovered in Ethiopia throughout the study [34]. The supplementary study showed that stunting and underweight were more common in males than females among east African children. This is consistent with the earlier Burkina Faso study [58]. One possible explanation is that, even after accounting for gestational phase and body size, boys still experience more complex childhood illnesses than females [62]. Children from mothers who had multiple births were more likely to have stunting and be underweight compared to singleton children at birth, which is similar to the Ethiopian research [24]. This finding further demonstrates the correlation between the wealth index and stunting and underweight. In line with the findings of an earlier study conducted in Bangladesh [33], a child from a low-wealth index household is more likely to be stunted and underweight. Increased revenue may be the cause, which boosts nutritional variety [63] and, in turn, boosts nutrient intake and nutritional status. This finding suggests that the risk that a kid would be stunted and underweight increases with the number of children under the age of five living in a household. This finding is consistent with the earlier research in Ghana [36]. Stunting and underweight were significantly influenced by the source of drinking water. Those from families without an improved source of water were more likely to have stunting and be underweight when compared to those who do. This study supports the conclusions of a prior investigation conducted in Ethiopia [34].

## Conclusion

The mother's education level, place of delivery, the size at birth, the age of the children, and the husband's education level were identified to be the common predictors that were significantly linked to the three undernourishment indices in this study. Common factors that were significantly associated with stunting and underweight included fever and diarrhea in the two weeks prior to the survey, the gender of the children, the type of birth the children had, the wealth status of the household, the number of children under the age of five in the household, the source of drinking water, and the location of residence. Family size and child birth order, however, were the only variables that were strongly linked with stunting. According to the study's findings, undernourishment among young children under the age of five continues to be a significant public health issue in the East African region. Governmental and non-governmental organizations should therefore plan public health engagement focusing on maternity and child mothers' husbands' education and the poorest households in order to improve children under the age of five's undernutrition status. Additionally, improving the delivery of healthcare at health facilities, places of residence, children's health education, and drinking water sources is essential for lowering child undernutrition indicators.

## Data Availability

The data used in this study are from the Measure DHS program https://dhsprogram.com/Data/terms-of-use.cfm, and can be accessed following the protocol outlined in the Methods section. Further documentations on ethical issues relating to the surveys are available at http://dhsprogram.com.

## Abbreviations

DHS: Demographic health survey
EAs: Enumeration areas
GDHS: Gambian demographic and health survey
Ref.: Reference Category
HAZ: Height for age standardized score
ICF: International Coaching Federation
WAZ: Weight for age standardized score
WHZ: Weight for height standardized score
OR: Odds ratio
UNDP: Untied Nation Development Program
